# Diversity of vaginal microbiota increases by the time of labor onset

**DOI:** 10.1038/s41598-017-17972-0

**Published:** 2017-12-14

**Authors:** Ekaterina Avershina, Silje Slangsvold, Melanie Rae Simpson, Ola Storrø, Roar Johnsen, Torbjørn Øien, Knut Rudi

**Affiliations:** 1 Department of Chemistry, Biotechnology and Food Science, University of Life Sciences, Ås, Norway; 2 Department of Public Health and Nursing, Norwegian University of Science and Technology, NTNU, 7491 Trondheim, Norway

## Abstract

Vaginal microbiota is an important early source of bacterial colonization for newborns. However, only a few small studies have investigated the composition of vaginal microbiota during labor. In this work, we analyzed vaginal swabs collected at 36 weeks gestation and at the onset of labor from 256 women participating in a randomized placebo-controlled study of probiotic supplementation for the prevention of atopic dermatitis in offspring. Although individuals’ vaginal microbiota was stable over time, several bacterial families, which are characteristic of mixed community state type (CST) IV, were overrepresented in vaginal swabs sampled at labor. Alpha-diversity also tended to increase by between 36 weeks gestation and the onset of birth. In the majority of women, CST remained the same throughout the study. Among the women who switched their vaginal microbiota from one CST to another, approximately half shifted towards CST IV. Although CST IV is often associated with bacterial vaginosis, which in turn may lead to preterm birth, in our cohort this shift was not associated with self-reported vaginosis, preterm delivery or birthweight. Probiotic consumption did not alter vaginal microbiota.

## Introduction

Vaginal microbiota of women can generally be separated into five vaginal bacterial community state types (CSTs) that seem to be dependent on ethnic and biogeographical background^1,2^. Four of the CSTs are characterized by high levels of either *Lactobacillus crispatus* (CST I), *Lactobacillus gasseri* (CST II), *Lactobacillus iners* (CST III) or *Lactobacillus jensenii* (CST V). On the other hand, CST IV lacks lactobacilli and is enriched in various anaerobic bacteria. Whilst CST IV can be found in healthy women, it is often linked to high Nugent scores and bacterial vaginosis^3^, which in turn is associated with increased risk of preterm birth and contraction of sexually transmitted infections^4^.

The vaginal microbiota plays a major role in initial colonization of the infant gut^5,6^. Disruption of this colonization due to, for example, Caesarean-section (C-section) may have an impact on gut microbial composition for years^7,8^, and these variations have been linked to the increasing rates of atopic disorders^9^. However, it seems that postnatal vaginal seeding of C-section born babies may result in a pattern of colonization that resembles what is observed in vaginally born babies^10^. It is therefore crucial to understand how vaginal microbial communities of laboring women are structured. Several large cohort studies have described vaginal microbiota during pregnancy^1,11,12^. However, vaginal microbiota may undergo rapid changes^13^ and we lack large studies of vaginal microbiota during labor. In fact, we could only find two studies that included women at labor^5,14^. In both of these studies, less than ten individuals were included.

In this work, we report bacterial composition of vaginal swabs from 256 women collected around 36 weeks gestation or at the time of admission to hospital during labor. Vaginal samples at the onset of labor were characterized by increased alpha-diversity and several bacterial families that are characteristic of CST IV were overrepresented. Among women who had a vaginal microbiota that changed from one CST to another, we also show that it was more common to switch to CST IV than to other CSTs. All women participated in a randomized double-blind placebo-controlled probiotic intervention study^15^ (the Probiotics in the Prevention of Allergy among Children in Trondheim; ProPACT), but the probiotic consumption did not lead to significant changes in vaginal microbiota composition.

## Methods

### Study cohort

The vaginal swab samples were collected at 36 weeks gestation and during labor from 256 women in the Probiotics in the Prevention of Allergy among Children in Trondheim (ProPACT) study^15^, Norway. In the ProPACT trial, participating women were randomized to receive a daily dose of fermented milk containing the three probiotic bacterial strains, or a placebo milk which was also fermented, contained no probiotic bacteria and was heat-treated after fermentation. Both the probiotic (n = 125) and placebo group (n = 131) were to consume 250 mL of their study milk per day from 36 weeks gestation until 3 months after birth. The probiotic milk contained *Lactobacillus rhamnosus* GG, *Lactobacillus acidophilus* La-5 and *Bifidobacterium animalis* subsp. *lactis* Bb-12. Eight women gave birth between 36^th^ and 37^th^ gestational week and the average birth weight of these babies was 3163 g (577 g) [mean (standard deviation SD)]. Characteristics of the study cohort are given in Table 1.

**Table 1:** Study cohort characteristics.

Characteristics	Treatment allocation			
	Probiotic		Placebo	
	n		n	
Mother’s age, yrs mean (SD)	125	30.4 (3.8)	131	30.3 (4.0)
Sex (male), child, n (%)	125	67 (53.6)	131	53 (40.5)
Birth weight, g mean (SD)	125	3692 (472)	131	3603 (491)
Siblings, n (%)	125	57 (45.6)	130	55 (42.3)
Atopy in family, n (%)	125	90 (72.0)	130	95 (73.1)
Maternal atopy, n (%)	125	58 (46.4)	129	65 (50.4)
Pet^a^, n (%)	125	34 (27.2)	130	36 (27.7)
Compliant^b^, n(%)	121	109 (90.1)	130	116 (89.2)

^a^Reported a household pet during pregnancy or the child’s first year of life; ^b^Compliance with the study protocol was defined as consumption of the study milk on at least 50% of days from 36 weeks gestation to 12 postpartum, no consumption of other products with probiotics and at least partial breastfeeding until 3 months postpartum.

The Regional Committee for Medical Research Ethics for Central Norway (Ref. 097-03, dated July 7, 2003), and the Norwegian Data Inspectorate (Ref. 2003⁄953-3 KBE⁄) approved the study and all participants signed an informed consent form. All experiments were performed in accordance with guidelines and regulations of The Regional Committee for Medical Research Ethics for Central Norway. The trial was registered in ClinicalTrials.gov (identifier NCT00159523, received September 8, 2005).

### Collection of samples

Bacterial swab samples during pregnancy were collected by the participating women and stored in their home freezer until they were transported to the study center and stored at −80 °C until analysis. The collection of samples during labor was done by midwives when the women presented to the labor ward and before they underwent any examinations. Both pregnancy and labor samples were collected on carbon impregnated cotton swabs and stored on Stuart Transport medium^16^.

### 16S rRNA gene dataset generation

For DNA extraction, samples were thawed on ice, vortexed and 100 µl of the solution was transferred to tubes containing 0.25 g acid-washed glass beads (<106 µm, Sigma Aldrich, USA) for mechanical lysis; 300 µl of the S.T.A.R. buffer (Roche, Germany) was also added to the tubes to prevent DNA degradation. Following the mechanical lysis step, DNA was isolated using a bead-based protocol and the V3-V4 region of the 16 S rRNA gene was sequenced in both directions as described previously^17^. Samples were processed on five 96-well plates and a negative control (no template) was included on each one of them.

### Sequencing data analysis

Sequencing reads were paired-end joined, filtered based on the quality score using QIIME^18^ and clustered at the 97% identity level with *usearch* v7.0^19^. Resulting operational taxonomic units (OTUs) were taxonomically classified by Greengenes database v13.8^20^. All further statistical analyses and diversity estimations were performed in MATLAB R2016b (MathWorks, USA).

Kruskal-Wallis non-parametric test was used for significance assessment and resulting p-values were corrected for multiple testing using Benjamini-Hochberg FDR correction^21^. Only OTUs with a relative abundance over 1% in at least one sample were used for significant difference assessments.

### Data availability

The dataset generated during the current study can be made available upon request to the corresponding author.

## Results

### General description of the vaginal microbiota

Three hundred and seventy-nine vaginal swab samples were available and all of these underwent DNA extraction. Samples that had failed DNA extraction or PCR amplification (n = 26) were not subjected to sequencing. We generated 5 261 752 paired-end joined sequencing reads from 353 samples with an average of 14 905 (SD = 9 286) reads per sample. The final dataset was rarefied to 3000 sequences per sample and comprised 335 samples which belonged to 256 women (n = 230 and n = 105 for 36^th^ gestation week and labor respectively). Seventy-nine women had adequate reads from both sampling time points.

**Figure 1:**
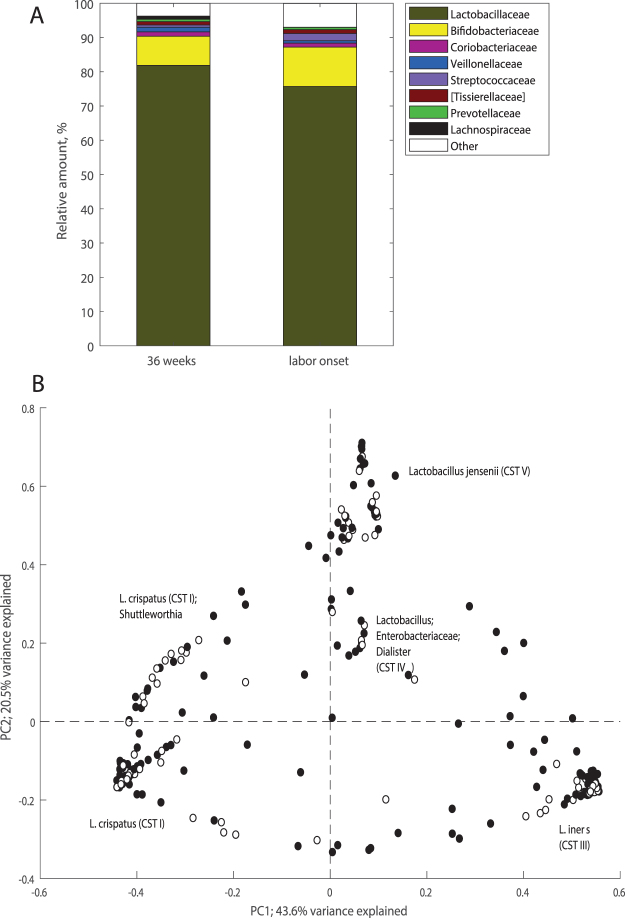
Microbial profiles of vaginal swab samples assessed by 16 S rRNA gene sequencing. (**A**) Average bacterial family composition. (**B**) PCoA clustering of samples based on Bray-Curtis dissimilarity index distance estimates, black and white circles represent samples from 36^th^ gestational week (n = 230) and labor (n = 105), respectively.

In total, 463 OTUs from 81 bacterial families were identified in the dataset, with OTUs from *Lactobacillaceae* and *Bifidobacteriaceae* families accounting for nearly 90% of the microbial population (Fig. 1A). On average, each woman was colonized by 3.8 (1.1) [mean (SD)] OTUs with Simpson’s reciprocal diversity index ranging from 1.0 to 22.7 (1.42 (0.98) [median (interquartile range IQR)]). Principal Coordinate Analysis (PCoA) revealed distinct clusters of the samples independent of sampling point (Fig. 1B). Dominance of *L. crispatus* (CST I) or *L. iners* (CST III) was the driving force of clustering majority of samples (n = 148 and n = 105 for the samples clustering into CST I and CST III respectively). *L. jensenii* (CST V) was characteristic of 28 samples. The remaining samples had lower abundance of these lactobacilli and were characterized by mixed communities of lactobacilli, Enterobacteriaceae, *Prevotella* and other bacteria (CST IV). Although *L. gasseri* was detected in some samples, none of them were dominated by this species and thus none of samples were classified as CST II. There were no associations between CST type at labor onset and birthweight of the child (FDR > 0.05 for each CST).

### Vaginal microbiota changes by the time of labor onset

Overall, the vaginal microbiota at labor was associated with a higher observed species index (3 (1) and 4 (2) [median (IQR)] for pregnancy and labor respectively; p = 1·10^−8^), but no difference in diversity was detected when taking evenness of species distribution into account (Simpson’s reciprocal diversity index). Eleven OTUs, none of which belonged to lactobacilli, had significantly higher levels at labor (FDR < 0.05 for each OTU; Supplementary Figure 1). On the family level, *Staphylococcaceae*, *Sphingomonadaceae*, *Pseudomonadaceae*, *Chitinophagaceae*, *Burkholderiaceae*, *Oxalobacteraceae* and *Comamonadaceae* tended to be overrepresented in vaginal swabs of women in labor (FDR < 0.05; Supplementary Figure 2).

**Figure 2:**
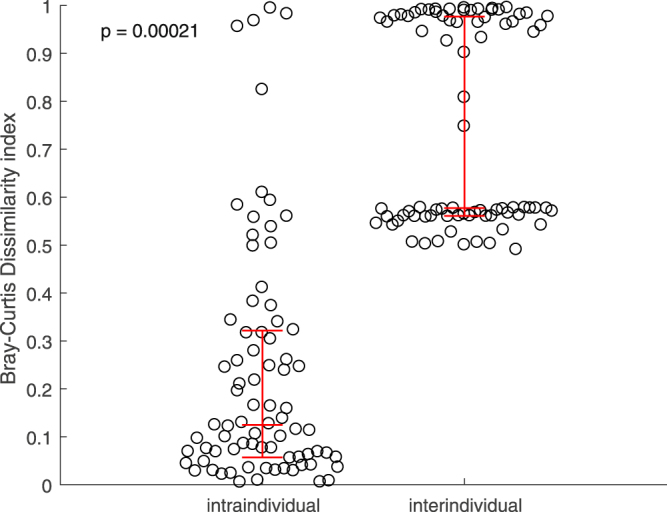
Intra- and inter-individual temporal variation of vaginal microbiota (158 samples from 79 women who had data on both time points). Red lines represent 25^th^, 50^th^ and 75^th^ quartiles.

**Figure 3:**
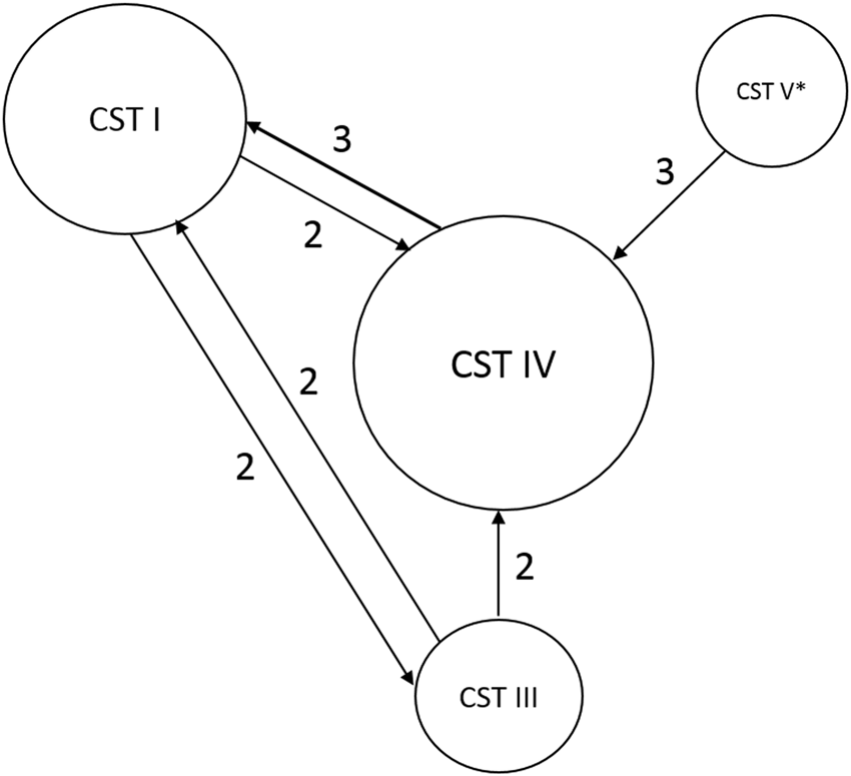
Schematic representation of the switch between CSTs between two sampling points. Fourteen women shifted their CST by the time of delivery. Number by the arrow represents number of women who switched to a given CST. Size of the circles reflects prevalence of a given CST by the time of delivery in fourteen women who changed CST type. *None of women remained CST V at delivery.

Among the women with data at both sampling points, their vaginal microbiotas were found to be more similar within rather than between individuals (Fig. 2). Indeed, only 14 of 79 women (17.7%) had a microbial composition that shifted from one CST type to another (Fig. 3). A switch to CST IV accounted for half of these changes.

Eighteen women had visible traces of blood in their vaginal swabs collected when they were admitted to hospital for labor. Overall, these samples did not have any significant difference in relative abundances compared to the blood-free samples. However, in 7 out of 11 women who had traces of blood and data on both time points, CST shifted by the onset of labor (63.3% vs 17.7% of the CST shift rate in the study population; binomial p = 9·10^−4^). Three women shifted towards CST IV, which was concordant with CST IV switch rate in the study population.

### Self-reported bacterial vaginosis during pregnancy and preterm birth were not associated with CST IV

Eight women reported having bacterial vaginosis or abnormal vaginal discharge during their pregnancy, and only three of these women indicated that symptoms were present in the 3^rd^ trimester (Table 2). Also, the majority of women with CST type IV report that they did not experience bacterial vaginosis or abnormal discharge at any point during pregnancy (Table 2).

**Table 2:** Frequency of self-reported bacterial vaginosis/unusual vaginal discharge and CST type during pregnancy and labor.

Bacterial vaginosis and CST type of pregnancy sample (n = 214)			
CST type	Never	Ever	In 3rd trimester
I	87	3	1
III	69	2	2
IV	30	2	0
V	20	1	0
**Bacterial vaginosis and CST type of labor sample (n = 97)**			
I	44	1	0
III	29	0	0
IV	17	2	1
V	4	0	0

There were also eight women in the cohort who gave birth prematurely (before week 37). None of these women reported bacterial vaginosis during pregnancy. One of them had CST I, four had CST III, and three had CST IV type vaginal microbiota at 36 weeks gestation.

### Probiotic supplementation does not alter vaginal microbial community

Probiotic supplementation did not have any significant effect on alpha-diversity (Simpson’s reciprocal diversity index 1.36 (0.83) and 1.45 (1.46) [median (IQR)] or observed species index 4 (2) and 4 (2) for probiotic and placebo groups, respectively; p > 0.05). Microbial abundance profiles of vaginal samples, both on OTU and family level, were also similar between the two groups (FDR > 0.05 between probiotic and placebo group for all OTUs and families). Since probiotic milk contained LGG, *L. acidophilus* La-5 and *B. animalis* subsp. *lactis* Bb-12, we searched all lactobacilli OTUs and bifidobacteria OTUs against BLAST NCBI 16 S ribosomal RNA sequences database. OTU39 gave closest hit to *Lactobacillus rhamnosus* (98% identity over 439 bp; E = 1·10^−166^) and none of the OTUs were identified either as *Lactobacillus acidophilus* or *Bifidobacterium animalis*. OTU39 was equally likely to be detected at probiotic and at placebo group during labor (6 out of 50 women in probiotic group and 6 out of 55 women in placebo group had detectable levels of OTU39 in their vaginal tracts; binomial p = 0.47).

### Low prevalence of Group B Streptococcus (GBS, *S. agalactiae*) in the dataset

OTU26 (*Streptococcus*) showed closest homology towards the reference genome of *Streptococcus agalactiae* (NC_004116; 99.3% identity over 433 bp; E = 1.49∙10^−155^). BLAST search of this OTU against NCBI 16 S ribosomal RNA sequence database revealed 19 additional streptococcal species that had at least 97% homology to this OTU and thus could have potentially been binned into the same OTU (Supplementary Table 1). We therefore aligned raw paired-end joined reads from the OTU26-positive samples against the 16 S rRNA gene sequence of *S. agalactiae* to address the specificity of this OTU. In total, there were 16 266 sequences with 99–100% pairwise identity to *S. agalactiae* 16 S rRNA gene. There was a highly significant correlation between relative amounts estimated based on OTU counts and on raw reads data (Pearson c = 0.9947; p = 3∙10^−39^; Supplementary Figure 3).

In total, *S. agalactiae* was detected in 34 women (13.2% of the population) and its abundance ranged from 0.03 to 30.1% (IQR = 1.9). We did not find any correlation between CST type frequencies in the study cohort and detection of OTU26 at either time point (binomial FDR > 0.05). Only six women were persistently colonized by this OTU throughout the study, and three of them have shifted the CST by the time of labor onset.

### Control for reagents contamination

Five negative controls included from the DNA extraction step, were also subjected to sequencing. Only one of them had sequencing reads that were not removed after quality filtering. This sample had 92 reads, most of which belonged to *Lactobacillus* (49 reads) and *Enterococcus* (18 reads) genera, possibly indicating cross-contamination of the negative control due to potential well-to-well spillage. The majority of other OTUs found in the negative control were detected as single reads (Supplementary Table 2). We marked these OTUs as ‘suspicious’ as they might have stemmed from the reagents contamination^22^. However, since only one out of five negative controls was amplified, and the number of sequencing reads in this sample was low, we did not exclude these OTUs from the dataset.

## Discussion

To our knowledge, our study is the first to describe the vaginal microbiota at the point of admission to hospital for labor in a large cohort of women (n = 105), as well as at the 36^th^ gestation week (n = 230). In concordance with previous observations, *L. crispatus* (CST I), *L. iners* (CST III) and *L. jensenii* (CST V) were the most commonly identified residents of the vaginal microbiota^3,4^. We found that the number of observed species increased significantly by the time of delivery. In particular, OTUs and bacterial families that are characteristic of CST IV were significantly overrepresented at labor compared to 36 weeks gestation. For women with samples at both time points (n = 79), the vaginal microbiota at labor generally had a greater resemblance to the pregnancy sample from the same woman than to other women. However, in 17.7% of our study cohort the CST shifted within the last weeks of pregnancy. In half of the women who changed the CST by the time of labor onset, the microbiota changed to CST IV. Although the tendency to switch from one CST to another during pregnancy has been previously reported, the shift to CST IV has rarely occured^1,3^. CST IV is often linked to high Nugent scores^3^, which are commonly used in assessment of bacterial vaginosis^23^. Although we do not have the Nugent scores for these samples, the frequency of self-reported vaginosis during pregnancy was low in our cohort and did not seem to be connected to any particular CST type. CST IV has been linked to increased risk of preterm birth^24^, but other studies do not find this association^12^. In our cohort, CST IV did not correlate to either preterm birth or birthweight of the child. Moreover, all women who shifted to CST IV by the time of labor onset, delivered at term. Increased rate of CST IV was also reported characteristic of 6-weeks-postpartum vaginal samples of women with uncomplicated term deliveries^1^. Since prenatal hormonal changes might contribute to the differences in the microbiota^25^, we believe that this can indicate that vaginal microbiota changes shortly before labor onset and then takes long time to be recovered back to its non-pregnant state. Women who had visible blood traces in their vaginal swab, tended to shift CST more often than other women, although a tendency towards CST IV shift in these samples was comparable to that of the whole study cohort.

We have previously demonstrated that mothers from the probiotic arm of the study had acquired the administered probiotic strains in their gut and their newborns were colonized by LGG^17,26^. Since it was mothers, and not their children, who received the probiotic supplement, it is fair to assume that LGG was transmitted from mother to child either at birth or during breast-feeding. However, we did not find any indication of LGG transfer via breast milk microbiota^27^ and the results of this study do not support the hypothesis of LGG translocation to the vaginal tract either. Another possible route of LGG transfer might be through oral microbiota via kissing or pacifiers, which are often cleaned by putting them in mothers’ mouths. Alternatively, since infants of LGG-colonized mothers were more likely to harbor this strain in their gut^17^, the colonization may occur through maternal gut microbiota.

Early-onset GBS (*S. agalactiae*) infection can be fatal for newborns and can cause neurologic impairment in the survivors^28^. Although we do not have access to medical records on maternal GBS status during labor, our sequencing data suggest rather low prevalence of *S. agalactiae* in vaginal swabs of women from our cohort (13%) compared to the average maternal colonization rate in Europe (19%)^29^.

The relative abundance of OTU56 (*Ralstonia*), which belongs to the *Burkholderiaceae* family, was found to be significantly different between pregnancy and labor onset. However, one read from this OTU was also detected in the negative control sample that had detectable sequencing reads. Taking into account that *Ralstonia* was previously reported as one of potential next-generation sequencing contaminants^22^, these results have to be taken with caution. We also report significant differences in *Chitinophagaceae*, which are commonly isolated from soil^30,31^. However, none of the reads in the negative control belonged to this bacterial family. Moreover, recent study on urinary microbiota has also detected *Chitinophagaceae* both in mid-stream urine and vaginal fluid samples^32^.

In conclusion, we report that although vaginal microbiota at labor resembled microbiota during pregnancy, bacteria associated with CST IV were significantly overrepresented. We also show that those women who shifted their dominant microbiota by the time of labor onset, tended to shift towards CST IV. CST IV microbiota type was not significantly associated with either self-reported bacterial vaginosis during pregnancy, preterm birth or birthweight of the child. Probiotic consumption did not affect the microbial community profile and administered probiotic strains were not translocated to the vaginal tract.

## Electronic supplementary material

Supplementary Information
