# Supplementary material for: Diversity of vaginal microbiota increases by the time of labor onset
Source: Sci Rep. 2017 Dec 14;7:17558. doi: 10.1038/s41598-017-17972-0 (PMC5730599; doi:10.1038/s41598-017-17972-0)
Supplement: Supplementary file 1 — Supplementary Information [file 41598_2017_17972_MOESM1_ESM.pdf]

Supplementary Information

'Diversity of vaginal microbiota increases by the time of labor onset'

Ekaterina Avershina[1*], Silje Slangsvold[1], Melanie Rae Simpson[2], Ola Storrø[2], Roar Johnsen[2], Torbjørn Øien[2] and Knut Rudi[1]

[1]Department of Chemistry, Biotechnology and Food Science, University of Life Sciences, Ås, Norway

[2]Department of Public Health and Nursing, Norwegian University of Science and Technology, NTNU, 7491 Trondheim, Norway

*corresponding author; email: ekaterina.avershina@nmbu.no; tel +47 6723 2447

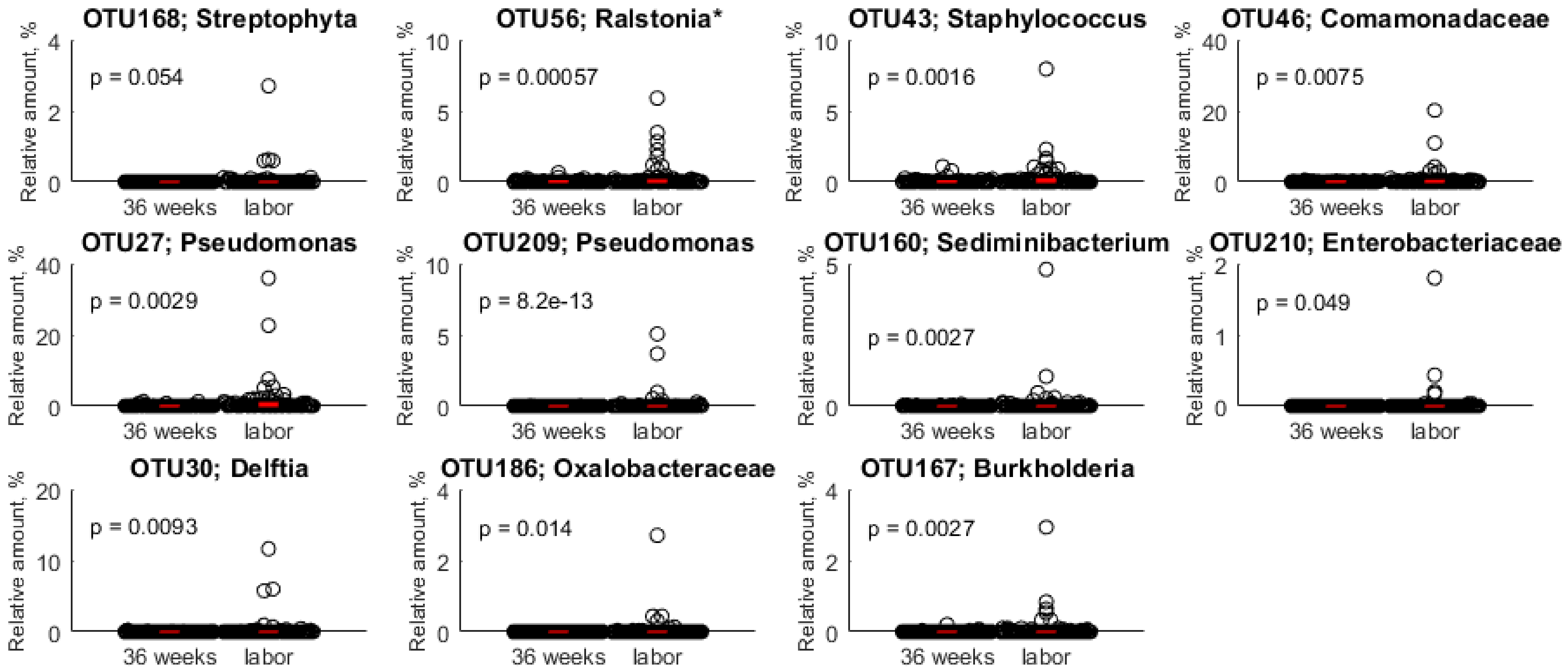

Supplementary Figure 1. OTUs that exhibit significant difference in relative abundance between 36th gestational week and delivery (FDR-corrected p-value is given in the upper left corner of each plot). Red lines represent 25th, 50th and 75th quartiles. *Reads belonging to OTU56 were detected in the negative control

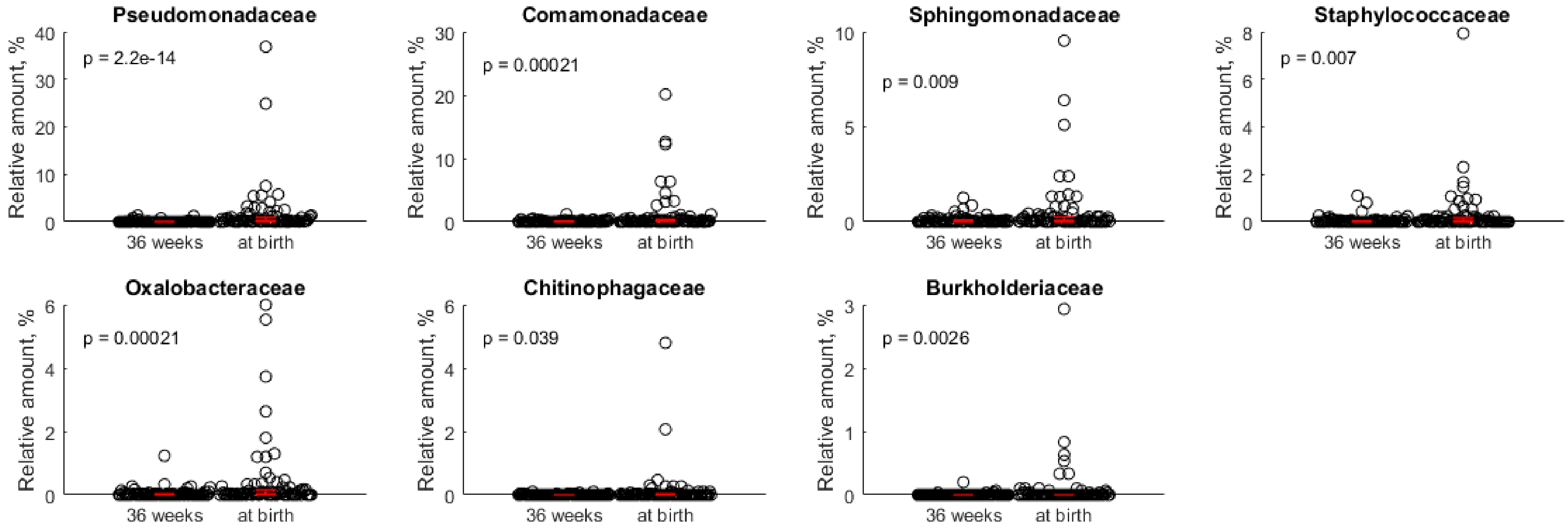

Supplementary Figure 2. Bacterial families that exhibit significant difference in relative abundance between 36th gestational week and delivery (FDR-corrected p-value is given in the upper left corner of each plot). Red lines represent 25th, 50th and 75th quartiles.

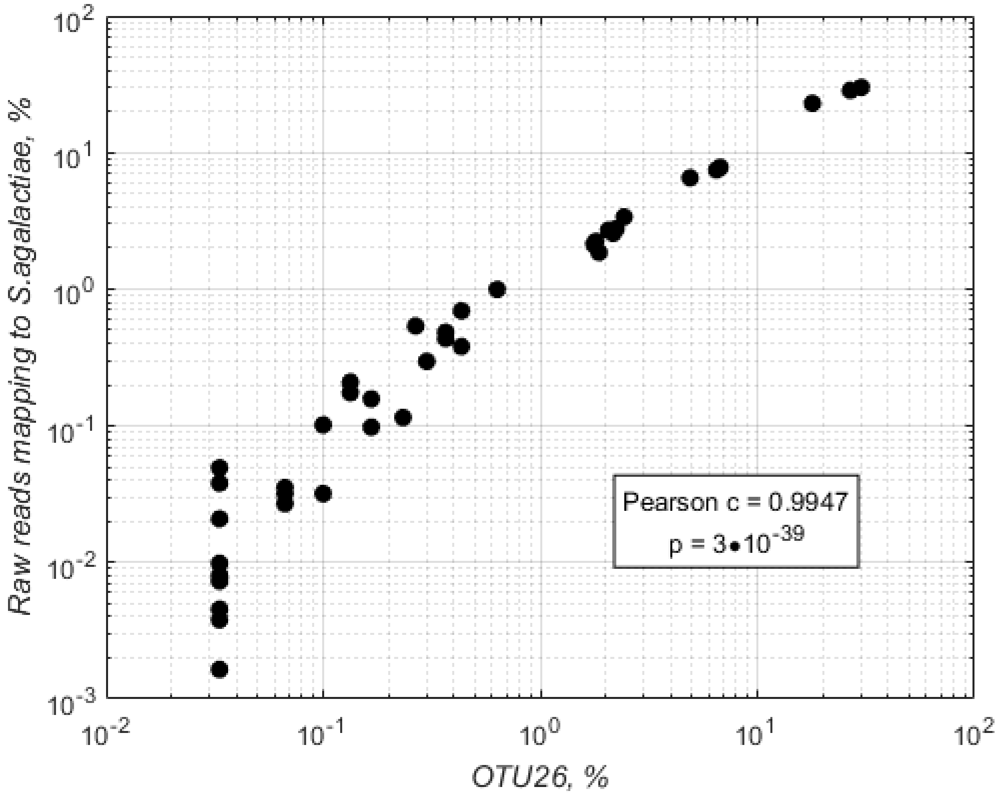

Supplementary Figure 3. Correlation between relative amounts of OTU26 (*Streptococcus*) and paired-end joined quality-filtered reads that exhibited ≥ 99 % homology towards *S. agalactiae* 16S rRNA gene

Supplementary Table 1. BLAST search of OTU26 (*Streptococcus*) against NCBI 16 ribosomal RNA database

Species	Number of hits	Mean E-value	Mean Identity, %
Streptococcus agalactiae	4	3E-167	99
Streptococcus dysgalactiae	6	2E-162	98.3
Streptococcus canis	2	7E-159	98
Streptococcus pyogenes	2	7E-159	98
Streptococcus urinalis	2	7E-159	98
Streptococcus halichoeri	1	3E-157	98
Streptococcus ictaluri	1	1E-160	98
Streptococcus equinus	3	7E-154	97
Streptococcus lutetiensis	3	7E-154	97
Streptococcus iniae	2	2E-155	97
Streptococcus alactolyticus	1	3E-152	97
Streptococcus caballi	1	3E-152	97
Streptococcus hyointestinalis	1	2E-155	97
Streptococcus infantarius	1	7E-154	97
Streptococcus macedonicus	1	3E-152	97
Streptococcus parauberis	1	7E-154	97
Streptococcus pasteurianus	1	3E-152	97
Streptococcus ratti	1	3E-152	97
Streptococcus ursoris	1	3E-152	97
Streptococcus equi	9	7E-154	97

Supplementary Table 2. OTUs detected in the negative control

OTUnum	Number of reads	Taxonomy
OTU_1	33	*Lactobacillus iners*
OTU_5	18	Enterobacteriaceae
OTU_2	11	*Lactobacillus*
OTU_100	7	Bifidobacteriaceae
OTU_84*	5	*Methylobacterium*
OTU_12*	4	Megasphaera
OTU_4	3	*Lactobacillus*
OTU_21*	2	*Clostridium*
OTU_3	2	*Lactobacillus*
OTU_11*	1	*Sneathia*
OTU_14*	1	*Prevotella*
OTU_30*	1	*Delftia*
OTU_36*	1	*Prevotella*
OTU_56*	1	*Ralstonia*
OTU_7	1	*Bifidobacterium*
OTU_9*	1	Coriobacteriaceae

*OTUs marked as ‘suspicious’
